# Progress in Medicine

**Published:** 1899-04-15

**Authors:** 


### Progress in Medicine.

### DISEASES OF THE THYROID GLAND.

**Myxœdema.**—Ord[1] has recently given an excellent description of the clinical features of myxœdema. The external appearance of the patients is much altered from the normal, there is an increase in the size and bulk of the whole body, due in part to changes in the skin, in part to changes in the subcutaneous tissue. The changes in the skin affect more or less the whole surface, and determine those in its appendages, in the glands, and in the organs of touch. The skin is everywhere dry and much thickened, not only in its dermal portion, but also in the epithelial part. It is very rough and harsh. The subepithelial tissues are swollen, but this swelling does not gravitate or pit, it is firm and resilient to the touch. The face, especially the eyelids and lips, is much swollen, so that the natural expression is lost, and the total effect is that of a mask of sorrowful immobility. Above the clavicles are often found soft projections beneath the skin, due partly to fat, partly to changed connective tissue. The hair all over the body dwindles and falls off, and the nails, though they rarely go so far as to complete atrophy, are wasted and brittle. All the visible mucous membranes exhibit alterations parallel with those of the skin. The cheeks project between the teeth and are marked by them, the tongue is very large, the uvula and soft palate press down upon the tongue as a somewhat firm, translucent, and imperfectly moveable mass. The teeth undergo impairment of nutrition, sometimes becoming brittle and sometimes coming out whole without much alteration in structure, whilst the gums are usually swollen and yet recede from the teeth, tending to bleed and ulcerate on the slightest provocation. Tactile sensation all over the body is diminished, due in part to the alteration of the connective tissue in and around nerve endings, but also to defect in the central receiving nervous system. This defect of sensation is, of course, particularly noticeable in parts such as the fingers, which depend for much of their usefulness on very delicate and sensitive nerves. As regards the muscular system there is in all cases a marked debility and tendency to ready exhaustion by exertion, sustained action of muscles evidently using up their power very readily. The want of co-ordination in the muscles may lead to sudden falls, and sometimes to severe injury. The speech of the myxœdematous patient is unlike any other modification of speech in disease. There is an obvious difficulty in getting words out of the mouth, in their ultimate pronunciation. The quality of the voice, too, is nasal and leathery, and the tone monotonous. The words uttered, although somewhat blurred, are usually correctly framed, and for the most part accurately represent what is in the mind. A curious feature is that, in spite of the slowness and painfulness of utterance, the patients once started in conversation tend to go on indefinitely. This tendency to garrulity persists after the mechanical obstacles have been removed by thyroid treatment. The aspect of the patients is lethargic, and a considerable number are actually lethargic and unnaturally placid; but it is very common to find indications of a disturbance of mental equilibrium. The commonest is the gradual development of a suspicious frame of mind, not a suspicion of conspiracy or intent to do harm, but a constant idea that people regard the patient unfavourably and are finding fault. This may make the patient actually violent, and to cause him to protest against the supposed injustice of the supposed attitude of the persons around. The mental disturbance may become so great as to require restraint. The temperature of the body is always lowered in well-developed cases from 1 deg. to 3 deg. Fahr. Related to this we find that patients are liable to great suffering and aggravation of their symptoms in cold weather. They do not appear so much to feel the cold as to be conscious of the injurious effects of cold. Hæmorrhages from various parts are not uncommon; thus epistaxis and bleeding from the gums often occur, and the extraction of a tooth is to be dreaded. Again, uterine hæmorrhage is often a serious source of enfeeblement, and cerebral hæmorrhage sometimes causes death. It is now generally admitted that the disease is dependent on a destruction or loss of function of the thyroid gland, and in some cases this may be preceded by a stage of hypertrophy, with or without the signs of exophthalmic goitre. The urine for the most part contains no albumen; certainly not, as a rule, until the disease has lasted for a very long time, often not at all from beginning to end. Where albumen is present regularly in the urine, together with casts, we may regard it as an indication that the kidney has undergone an internal change comparable to that observable in the eyelids.

Murray,[2] in his Goulstonian lectures, gives a *résumé*

of recent work on the parathyroid glands. These glands, which are composed of solid cylinders of cells, devoid of any central lumen like the thyroid proper, are in some animals embedded in the thyroid; in others, the outside of it. Gley showed that in rabbits removal of the parathyroid glands along with the thyroid produces different effects from those due to removal of the thyroid only. The double operation is followed by acute symptoms, the most important of which are muscular tremors commencing in the masseter muscles, clonic and often tetanic contractions of the voluntary muscles, paralysis of the fore feet followed by that of the hind limbs, difficult and often rapid respiration, salivation, dilatation of the pupils, and rise of temperature. On the other hand, removal of the thyroid gland only is followed by no such immediate effects, but after a long interval a chronic cachexia develops which closely resembles myxœdema in man.

In animals, such as monkeys, it is not possible to remove the thyroid or parathyroid only; the latter is so embedded in the thyroid. In such cases it is found that after experimental thyroidectomy (*i.e.*, where both are removed) the administration of thyroid gland, while it produces a marked benefit, does not save the animal's life as a rule; it dies with acute nervous symptoms such as those mentioned above. These results are in harmony with those of Gley's. And they have also been confirmed by Moussu[3] and by Charrin.[3] The former found that, if both thyroid and parathyroid were excised and acute nervous symptoms had commenced, the injection of an extract of parathyroid led to a disappearance of the spasms. And, conversely, Charrin found that the administration of parathyroid did not remove the symptoms of myxœdematous patients.

[1] Ord, Lancet, Nov. 12, 1898. [2] Murray, Lancet, Mar. 11 and 18, 1899. [3] Moussu and Charrin, Le Semaine Médicale, Aug. 3, 1898.

